# A rehabilitation robot control framework with adaptation of training tasks and robotic assistance

**DOI:** 10.3389/fbioe.2023.1244550

**Published:** 2023-10-02

**Authors:** Jiajun Xu, Kaizhen Huang, Tianyi Zhang, Kai Cao, Aihong Ji, Linsen Xu, Youfu Li

**Affiliations:** ^1^ College of Mechanical and Electrical Engineering, Nanjing University of Aeronautics and Astronautics, Nanjing, China; ^2^ College of Mechanical and Electrical Engineering, Hohai University, Changzhou, China; ^3^ Department of Mechanical Engineering, City University of Hong Kong, Kowloon, Hong Kong SAR, China

**Keywords:** rehabilitation robotics, human-robot interaction, biological signal, trajectory deformation, assist-as-needed control

## Abstract

Robot-assisted rehabilitation has exhibited great potential to enhance the motor function of physically and neurologically impaired patients. State-of-the-art control strategies usually allow the rehabilitation robot to track the training task trajectory along with the impaired limb, and the robotic motion can be regulated through physical human-robot interaction for comfortable support and appropriate assistance level. However, it is hardly possible, especially for patients with severe motor disabilities, to continuously exert force to guide the robot to complete the prescribed training task. Conversely, reduced task difficulty cannot facilitate stimulating patients’ potential movement capabilities. Moreover, challenging more difficult tasks with minimal robotic assistance is usually ignored when subjects show improved performance. In this paper, a control framework is proposed to simultaneously adjust both the training task and robotic assistance according to the subjects’ performance, which can be estimated from the users’ electromyography signals. Concretely, a trajectory deformation algorithm is developed to generate smooth and compliant task motion while responding to pHRI. An assist-as-needed (ANN) controller along with a feedback gain modification algorithm is designed to promote patients’ active participation according to individual performance variance on completing the training task. The proposed control framework is validated using a lower extremity rehabilitation robot through experiments. The experimental results demonstrate that the control scheme can optimize the robotic assistance to complete the subject-adaptation training task with high efficiency.

## 1 Introduction

Due to the rapidly increasing number of physically and neurologically impaired patients around the world, rehabilitation robots have been developed to assist in the therapeutic training of impaired limbs, which improves rehabilitation efficiency and saves human labor through highly autonomous assistance (Xu et al., 2020a; Xu et al., 2020b). The control strategy of a rehabilitation robot significantly influences rehabilitation efficacy. Most clinical cases enable physiotherapists to feed the task trajectories into the robot controller before rehabilitation starts. However, patients can only modify the robot’s current trajectory through physical human-robot interaction (pHRI) without affecting the future task trajectory. Particularly for patients with severe impairment, it is hardly possible to continuously exert adequate force to change the robot’s movement trajectory for a period, and their recovery, comfort, and safety cannot be guaranteed accordingly. Therefore, online adaptation of desired trajectories to patients’ performance is indeed necessary. Dynamic movement primitive (DMP) (Schaal, 2006) and central pattern generator (CPG) (Sproewitz et al., 2008) are two common tools for trajectory generation, and they have been applied in rehabilitation robotics research (Luo et al., 2018; Yuan et al., 2020). In (Xu et al., 2023), coupled cooperative primitives are formulated, where pHRI is expressed as a first-order impedance model and assumed as a modulation term in DMP. The problems existing in tuning the impedance model parameters have been explained above. The human-robot interaction energy is combined with adaptive CPG dynamics to plan gaits for exoskeletons (Sharifi et al., 2021); however, this introduces many uncertain parameters, the resolution of which is time-consuming. In addition, it is found that the robot’s desired trajectory can be deformed in response to subject actions (Lasota and Shah, 2015), but it is unclear which deformed trajectory is optimal. The robot’s future desired trajectory can be modified, and the optimal solution of the trajectory deformation is derived by detecting the human-robot interaction force (Losey and O’Malley, 2018). Similarly, trajectory deformation is applied to robotic rehabilitation, where a position controller is adopted to track the deformed trajectory, ignoring the rehabilitation effectiveness of the AAN training (Zhou et al., 2021). Apart from movement trajectory planning, users’ voluntary participation should be stimulated by adjusting the robotic assistance.

Additionally, passive control is usually employed to drive impaired limbs to move along the predefined task trajectory, where the active participation of patients cannot be encouraged to stimulate motor function recovery (Hogan et al., 2006). To overcome this problem, the assist-as-needed (AAN) control strategy is introduced to adapt the robotic assistance to the patients’ performance (Marchal-Crespo and Reinkensmeyer, 2009). An impedance/admittance control scheme is a common solution for addressing the physical relationship between humans and robots. An impedance controller based on a virtual tunnel regulates the robotic assistance while responding to the tracking error between the current trajectory and the desired trajectory (Krebs et al., 2003). A torque tracking impedance controller is proposed for lower limb rehabilitation robotics to generate assistance while ensuring acceptable trajectory deviation (Shen et al., 2018). An admittance control incorporating electromyography (EMG) signals is developed to improve human-robot synchronization (Zhuang et al., 2019). Both impedance and admittance control can regulate the relationship between the trajectory deviation and interaction effect by tuning the inertia-damping-stiffness parameters. In fact, different patients or even the same patient at different rehabilitation stages can exhibit varying motor capabilities; so, accurate determination of these parameters is essential to realize AAN training for different subjects and tasks. Furthermore, as for practical application in rehabilitation, dynamic human force and uncertain external disturbances often occur and cannot be measured intuitively and accurately. Both inappropriate impedance/admittance parameters and unknown dynamic interactive environments can lead to unstable and oscillating robot behaviors, which may decrease motion smoothness and even threaten human safety (Ferraguti et al., 2019). Furthermore, current AAN controllers are designed to provide only the necessary robotic assistance to complete the prescribed training task, which is not suitable for encouraging patients to challenge themselves with more difficult tasks and stimulate their potential motor capabilities for improved rehabilitation efficacy.

In this article, a control framework is proposed for the simultaneous adaptation of training tasks and robotic assistance according to patient performance. The main contributions of this article are listed as follows.

(1) A trajectory deformation algorithm is developed to plan the robot’s desired trajectory as the high-level controller, where the continuity, smoothness, and compliance of the robotic motion are achieved in response to the subject’s biological performance.
(2) An AAN control strategy with a feedback gain modification algorithm is employed to regulate the robotic assistance as the low-level controller. The controller is designed to encourage active participation by learning the patient’s residual motor capabilities and accurately tracking the deformed trajectory.
(3) Both the training task and robotic assistance adaptation algorithms are integrated into a framework to realize human-in-the-loop optimization, and this control framework is validated using a lower extremity rehabilitation robot.

The remainder of the article is organized as follows. The biological signal processing is described in Section 2. The trajectory generation is presented in Section 3, and the subject-adaptive AAN controller is explained in Section 4. The proposed control framework is verified through experiments in Section 5. Finally, this study is concluded in Section 6.

**FIGURE 1:**
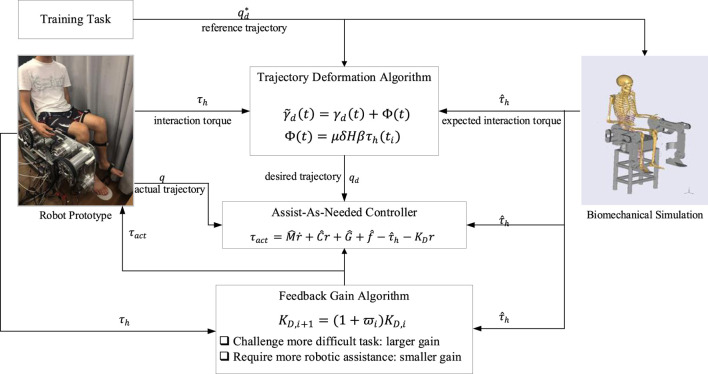
Control framework of simultaneous adaptation of training tasks and robotic assistance.

The schematic view of the proposed control framework is presented in Figure 1, and the detailed explanation is elaborated in the following sections.

## 2 Biological signal processing

For rehabilitation, robotic motions should be regulated by adapting to the user’s muscle strength, which can be derived from the human skin surface EMG signals. In this section, the EMG-driven musculoskeletal model for torque estimation is presented. EMG sensors (ETS FreeEMG300) are used to measure EMG signals, and the electrodes are attached to the relevant skin surface. Specifically, in the application of lower extremity rehabilitation, six electrodes are attached to gluteus maximus, semimembranosus, biceps femoris iliopsoas, sartorius, and rectus femoris for hip flexion/extension; six electrodes are attached to rectus femoris, vastus medialis, vastus lateralis, biceps femoris, semimembranosus, and semitendinosus for knee flexion/extension. The raw EMG signals are sampled at 1,024 Hz, bandpass filtered from 10 Hz to 500 Hz, and then notch filtered at 50 Hz to remove noise. The muscle activation is calculated by the neural activation function as

$$a\left(u\right)=\frac{e^{AuR^{-1}}-1}{e^{A}-1}$$ (1)

where u is the post-processed EMG value, *R* is the maximum voluntary isometric contraction, and A<0 is a nonlinear shape factor, defining the curvature of the function.

Subsequently, a Hill-typed muscle model is constructed to describe the relationship between muscle activation and muscle force (Yao et al., 2018). The force produced by the muscle-tendon unit $F_{mt}$ is given by the following set of equations:

$$F_{mt}=F_{m}\cos\emptyset =\alpha \left(F_{pe}+F_{ce}\right)\cos\emptyset$$ (2)

$$l_{mt}=l_{t}+l_{m}\cos\emptyset$$ (3)

where $F_{mt}$ , $F_{m}$ , $F_{ce}$ , and $F_{pe}$ represent the force generated by the muscle-tendon unit, the tendon force, contractile element, and passive element, respectively; $l_{t}$ is the length of the muscle tendon; α is a scaling factor; ∅ is the pinnation angle that is given by

$$\emptyset =\arcsin\left(\frac{\sin\left(\emptyset_{0}\right)}{l_{0}}\right)$$ (4)

where $\emptyset_{0}$ is the optimal pinnation angle and $l_{0}$ is the optimal length of muscle fiber. The scale factor α will be used in the optimization process. The human joint torque was produced by the coupling function of both the agonistic and antagonistic muscles, that is,

$${\hat{\tau }}_{h}={\left|\sum_{i=1}^{J}\tau_{i}\right|}_{agonist}-{\left|\sum_{j=1}^{L}\tau_{j}\right|}_{antagonist}$$ (5)

where $\tau_{i}=F_{i}^{m}r_{i}^{m}$ and $\tau_{j}=F_{j}^{m}r_{j}^{m}$ denote the torques exerted by the agonistic and antagonistic muscles, respectively. $F_{i}^{m}$ and $F_{j}^{m}$ are the muscle-tendon forces, $r_{i}^{m}$ and $r_{j}^{m}$ are the muscle moment arms of the muscle-tendon unit and can be estimated by determining the muscle-tendon length $l_{mt}$ and joint angle *q*; that is, $r^{m}=\frac{\partial l_{mt}}{\partial q}$ . The parameters *J* and *L* denote the number of agonistic and antagonistic muscles acting on the joint, respectively.

In the proposed EMG-driven musculoskeletal model, it is essential to determine the model parameters, i.e., the shape factor A and scaling factor α . Furthermore, the human joint torque during the exercise could be directly detected via AnyBody Modeling System (AMS) (Damsgaard et al., 2006). However, the torque generated by AMS is not continuous and realistic. The real joint torque can be measured through calibration experiments with EMG signals, which are quite complex and time-consuming. Therefore, it is proposed that the optimization is undertaken to adjust the EMG-driven musculoskeletal model parameters to minimize the difference between the torque estimated by the EMG signals ${\hat{\tau }}_{h}$ and the torque detected via AMS $\tau_{AMS}$ .

**FIGURE 2:**
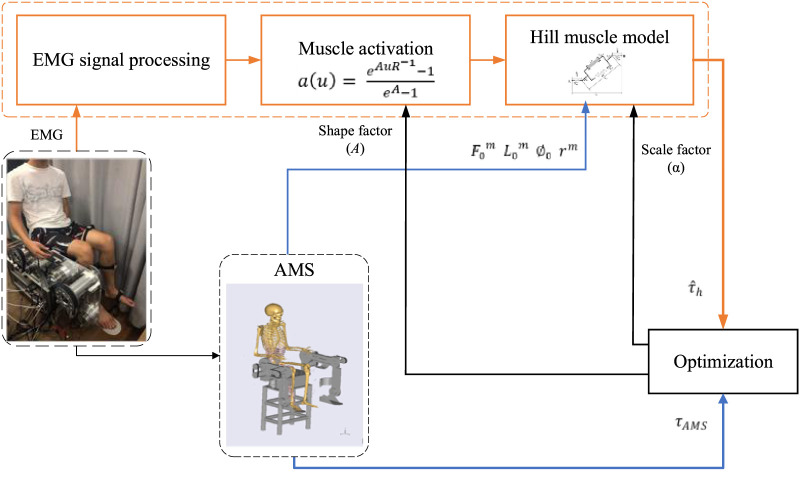
Optimization procedure of pHRI estimated from biological signals.

The optimization procedure is illustrated in Figure 2. The processed EMG signals are converted to muscle activation using (2) including the uncertain shape factor *A*. Then the muscle contraction model (Yao et al., 2018) is established to calculate the muscle-tendon force and the muscle torque through Equations 2–5, where the muscle-tendon length $l_{mt}$ and the moment arm $r^{m}$ can be obtained from AMS. On the other hand, certain driving motion is loaded to AMS and the human joint torque $\tau_{AMS}$ can be achieved. The optimization aims to shrink the difference between ${\hat{\tau }}_{h}$ and $\tau_{AMS}$ . Select the parameters to be optimized as $p={\left[\begin{array}{cc} A & \alpha \end{array}\right]}^{T}$ . The optimization problem is defined as (6).

$$\min J\left(p\right)=\sum_{i=1}^{N}\frac{{\left({\hat{\tau }}_{h}^{i}-\tau_{AMS}^{i}\right)}^{2}}{N}$$ (6)

where *N* is the number of samples. The Broyden–Fletcher–Goldfarb–Shanno algorithm (Peña et al., 2019), together with a penalty barrier algorithm, is employed to find the optimal parameters.

## 3 Trajectory adaptation

Prior to operating the rehabilitation robot, the training task needs to be predetermined by feeding the reference trajectory (task trajectory) into the robot controller, which is usually the natural gait trajectory of healthy subjects. The patient is then encouraged to complete the task with robotic assistance. Once the reference trajectory is preset and fed into the robot controller, however, it is not reasonable to maintain the task difficulty invariant throughout the rehabilitation procedure. In order to ensure the smoothness and compliance of the robotic motion, the robot’s desired trajectory should be modified intuitively and continuously in response to the human force (Losey and O’Malley, 2018). In this regard, the physical human-robot interaction (pHRI) should alter not only the robot’s current state but also its future behavior. In this section, a trajectory deformation algorithm is studied to explore the pHRI influence on the training task, and the modification is made on the original reference trajectory, generating the desired trajectory for the further controller design.

**FIGURE 3:**
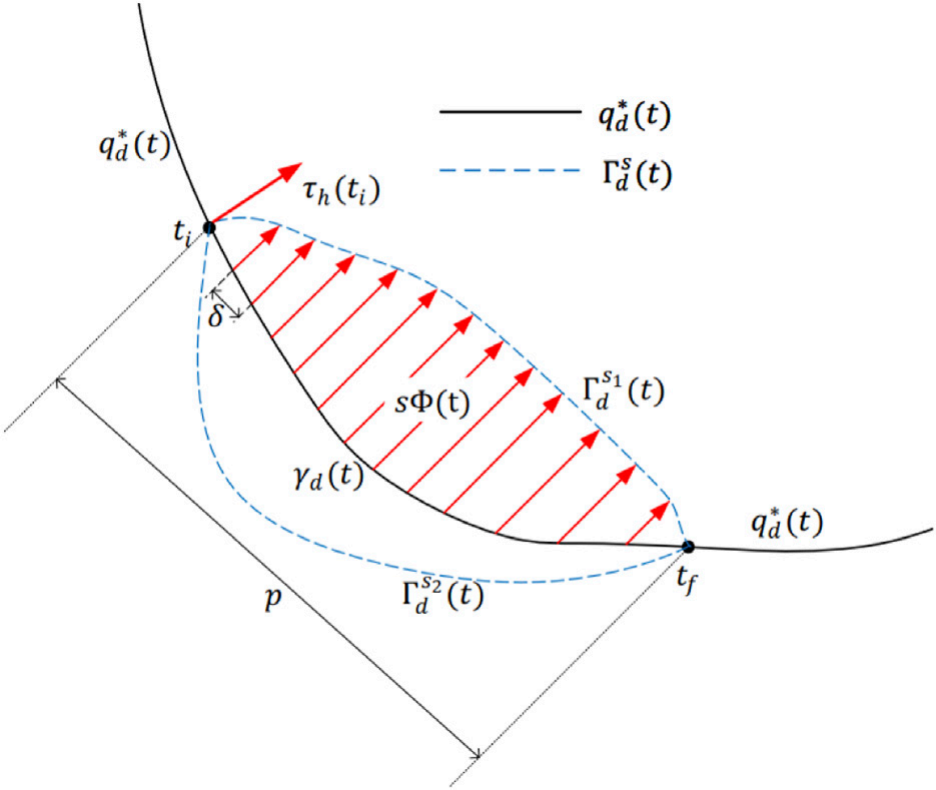
Diagram of the trajectory deformation.

The reference trajectory (predetermined before the training) is defined as $q_{d}^{*}$ , and the desired trajectory (altered during the training) is defined as $q_{d}$ . When the human-robot interaction torque $\tau_{h}$ is exerted on the robotic joints at time $t_{i}$ , the original desired trajectory $q_{d}^{*}$ starts to deform; such trajectory deformation ends at time $t_{f}$ , and accordingly, the duration of the trajectory deformation is $p=t_{f}-t_{i}$ . Moreover, the deformed trajectory between $t_{i}$ and $t_{f}$ can be evenly divided into an arbitrary number of waypoints, and the time interval between the consecutive waypoints is δ . Concisely, the deformation process can be expressed as $q_{d}^{*}\left(t\right)\to q_{d}\left(t\right)$ , where $t\in \left[t_{i},t_{f}\right]$ , and a diagram of the trajectory deformation is presented as Figure 3. It can be seen that both the magnitude and direction of the human-robot interaction torque influence the shape of the deformed trajectory. The larger the force exerted on the robot, the greater the deviation between the original and deformed trajectories; conversely, smaller human force results in smaller trajectory change. Besides, human force with opposite direction can lead to alteration in the deformation direction. So, both the amplitude and direction of the trajectory deformation should be taken into consideration while proposing the trajectory adaptation method.

Apparently, deformed from the reference trajectory may follow different curves to shape $q_{d}$ , and there are many possible trajectory deformations. Constrained over the time interval $\left[t_{i},t_{f}\right]$ , $\Gamma_{d}^{s}\left(t\right)$ is defined as the deformation curve function, in which s is the deformation factor, and changing the value of s can derive different shapes of trajectories. When s=0 , the segment of the desired trajectory between times $t_{i}$ and $t_{f}$ is represented as

$$\gamma_{d}\left(t\right)=\Gamma_{d}^{0}\left(t\right)\forall t\in \left[t_{i},t_{f}\right]$$

$$\gamma_{d}=\left[q_{d}^{*}\left(t_{i}\right),q_{d}^{*}\left(t_{i}+\delta \right)\ldots \;q_{d}^{*}\left(t_{f}-\delta \right),q_{d}^{*}\left(t_{f}\right)\right]$$ (7)

where $\gamma_{d}\left(t\right)=q_{d}^{*}\left(t\right)$ when $t\in \left[t_{i},t_{f}\right]$ , and $\gamma_{d}$ is not defined outside this time interval. All other values of s refer to deformations of $\gamma_{d}$ . As shown in Figure 3, in the time interval $\left[t_{i},t_{f}\right]$ , the original trajectory $\gamma_{d}\left(t\right)$ can be deformed to $\Gamma_{d}^{s_{1}}\left(t\right)$ and $\Gamma_{d}^{s_{2}}\left(t\right)$ .

When the subject starts to exert force at time $t_{i}$ , the robot’s desired trajectory is changed from $\gamma_{d}$ to ${\tilde{\gamma }}_{d}$ when s=1 , which is defined as

$${\tilde{\gamma }}_{d}\left(t\right)=\Gamma_{d}^{s}\left(t\right)$$

$${\tilde{\gamma }}_{d}=\left[\Gamma_{d}^{s}\left(t_{i}\right),\Gamma_{d}^{s}\left(t_{i}+\delta \right)\ldots \Gamma_{d}^{s}\left(t_{f}-\delta \right),\Gamma_{d}^{s}\left(t_{f}\right)\right]$$ (8)

Once ${\tilde{\gamma }}_{d}$ is determined, the robot’s desired trajectory is updated as $q_{d}\left(t\right)={\tilde{\gamma }}_{d}\left(t\right)$ . After time $t_{f}$ , the robot follows its reference trajectory $q_{d}^{*}$ again.

Comparing (7) with (8), the vital factor that causes the trajectory deformation can be formulated as a vector field function $\Phi \left(t\right)$ , which linearizes the dependency of $\Gamma_{d}^{s}\left(t\right)$ on the deformation factor s , i.e.,

$$\Gamma_{d}^{s}\left(t\right)=\gamma_{d}\left(t\right)+s\Phi \left(t\right)$$ (9)

The vector field function $V\left(t\right)$ is distributed along $\gamma_{d}$ and yields that $\Phi \left(t\right)=\partial_{s}\Gamma_{d}^{0}\left(t\right)$ , $\forall t\in \left[t_{i},t_{f}\right]$ , in particular, when s=1 , ${\tilde{\gamma }}_{d}=\gamma_{d}+\Phi \left(t\right)$ . The determination of $\Phi \left(t\right)$ should ensure the continuity, smoothness, and compliance of the deformed trajectory. Specifically, the transition from $q_{d}^{*}\left(t_{i}\right)$ to $q_{d}\left(t_{i}\right)$ and from $q_{d}\left(t_{f}\right)$ to $q_{d}^{*}\left(t_{f}\right)$ should be as continuous as possible. Also, a minimum-jerk model (Li et al., 2017) is utilized to generate a smooth trajectory profile to guarantee patients’ comfort and security. The vector field function $\Phi \left(t\right)$ is highly correlated to the human-robot interaction torque $\tau_{h}$ , and a cost function is designed and minimized to optimize the deformed trajectory for high compliance. The detailed explanation of determining the vector field function is presented in Appendix 1, and the resultant formulation of the vector field function is obtained as

$$\Phi \left(t\right)=\mu \delta H\beta \tau_{h}\left(t_{i}\right)$$ (10)

where

$$H=\frac{G}{\left(p+\delta \right)\left\|G\right\|}$$ (11)

$$G=\left(I-{\left(A^{T}A\right)}^{-1}B^{T}{\left(B{\left(A^{T}A\right)}^{-1}B^{T}\right)}^{-1}B\right){\left(A^{T}A\right)}^{-1}$$ (12)

The parameter $I\in R^{N\times N}$ in (12) is an identity matrix, where N denotes the number of waypoints. The determination of the matrix A and B is introduced in Appendix 1. The parameter H influences the shape of Φ , and it is formulated in (11). The parameter $\beta \in R^{N}$ in (10) is the prediction vector of the interaction torque, i.e., the future interaction torque can be formulated as $\beta \tau_{h}\left(t_{i}\right)$ , with $\tau_{h}\left(t_{i}\right)$ being the interaction torque applied at time $t_{i}$ . The direction of the interaction torque is included in the prediction vector so that the proposed algorithm can address the magnitude and direction of the trajectory deformation well. Even so, when the human force direction is altered, the deformed trajectory is suggested to be recalculated with the updated prediction vector for delicate modulation of the training task. Besides, in robotics-assisted rehabilitation, the duration of pHRI is relatively long because the patient mostly tries to participate actively to guide the robot, which is different from the statement in (Losey and O’Malley, 2018). The parameter μ in (10) denotes the assistance level, and it can be tuned to arbitrate between human and robot. When μ increases, the induced trajectory deformations arbitrate toward the human, which means smaller input forces cause larger deformations; and *vice versa*. Herein, the assistance level is formulated as $\mu =\tau_{h}/{\hat{\tau }}_{h}$ , where ${\hat{\tau }}_{h}$ is the expected human joint torque to complete the task trajectory in the absence of robotic assistance, and it can be obtained from Section 2.

Combining (9), (10), (11), and (12), the relationship between the vector field function and the interaction torque is clarified, and the deformed trajectory is thus obtained as

$${\tilde{\gamma }}_{d}=\gamma_{d}+\mu \delta H\beta \tau_{h}\left(t_{i}\right)$$ (13)

After ${\tilde{\gamma }}_{d}$ is derived from (13), $q_{d}$ is updated to include ${\tilde{\gamma }}_{d}$ , and the process iterates at the next trajectory deformation when pHRI occurs again.

## 4 Assist-as-needed controller

Based on the abovementioned trajectory generation scheme, an actuation controller needs to be designed to track the desired trajectory. More importantly, in response to various motor capabilities of different patients, a subject-adaptive controller is required to realize AAN training. In this section, an AAN control strategy along with a feedback gain modification algorithm is proposed to complete the training task motion and provide the minimum required assistance to encourage patients’ active engagement.

The robot dynamics in joint space can be presented as follows whilst considering pHRI.

$$M\left(q\right)\ddot{q}+C\left(q,\dot{q}\right)\dot{q}+G\left(q\right)+f\left(\dot{q}\right)+\tau_{dis}=\tau_{act}+\tau_{h}$$ (14)

where $q\in R^{n}$ ( n denotes the number of the robotic joints) is the position coordination of the robotic joints, and accordingly, $\dot{q}$ and $\ddot{q}$ denote the joint velocity and acceleration, respectively. The parameter $M\left(q\right)\in R^{n\times n}$ is the inertia matrix, $C\left(q,\dot{q}\right)\in R^{n\times n}$ is the centripetal and Coriolis matrix, $G\left(q\right)\in R^{n}$ is the gravity torque, $f\left(\dot{q}\right)\in R^{n}$ is the friction, $\tau_{dis}\in R^{n}$ is the external disturbance, $\tau_{act}\in R^{n}$ is the robotic joint torque generated by the actuators, and $\tau_{h}\in R^{n}$ is the human-robot interaction torque.

Although the robot dynamics have been modeled as (14), it is impossible to accurately formulate disturbances that may decrease the compliance of robotic motion and the safety of pHRI. The total disturbances $\tau_{d}$ include the estimation error of the human force ( $\tau_{h}-{\hat{\tau }}_{h}$ ), external disturbance ( $\tau_{dis}$ ) and unmodeled dynamics. So, the dynamics (14) can be rewritten as

$$\hat{M}\left(q\right)\ddot{q}+\hat{C}\left(q,\dot{q}\right)\dot{q}+\hat{G}\left(q\right)+\hat{f}\left(\dot{q}\right)={\hat{\tau }}_{h}+\tau_{act}+\tau_{d}$$ (15)

where $\hat{M}\left(q\right)$ , $\hat{C}\left(q,\dot{q}\right)$ , $\hat{G}\left(q\right)$ , and $\hat{f}\left(\dot{q}\right)$ are the estimation of $M\left(q\right)$ , $C\left(q,\dot{q}\right)$ , $G\left(q\right),$ and $f\left(\dot{q}\right)$ , respectively; $\tau_{d}\in R^{n}$ denotes the total disturbances. The position tracking error is defined with respect to the desired trajectory as $\tilde{q}=q-q_{d}$ , and the sliding variables are defined as

$$e=\dot{\tilde{q}}+\Lambda \tilde{q}$$ (16)

where Λ is a constant. In order to help subjects complete the desired tasks while providing the minimum required assistance, the AAN controller for the robotic actuation can be presented as

$$\tau_{act}=\hat{M}\dot{e}+\hat{C}e+\hat{G}+\hat{f}-{\hat{\tau }}_{h}{-K}_{D}e$$ (17)

where $\hat{M}$ , $\hat{C}$ , $\hat{G}$ , and $\hat{f}$ are the estimation of $M\left(q\right)$ , $C\left(q,\dot{q}\right)$ , $G\left(q\right),$ and $f\left(\dot{q}\right)$ , respectively, and $K_{D}\in R^{n\times n}$ is a positive-definite feedback gain. The selection of the parameter $K_{D}$ will be elaborated in the subsequent feedback gain modification algorithm.

Through the stability analysis in Appendix 1, we can conclude that the proposed control system yields a tracking error with uniformly ultimately bounded stability. The ultimate bound on the tracking error e can be expressed as $B_{e}$ , and its formulation is given in Appendix 1. Should $\tilde{M}\to 0$ , $\tilde{C}\to 0$ and $\tau_{d}\to 0$ , the appended analysis demonstrates that $\left\|e\right\|\to 0$ , and the system proves globally asymptotically stable. The inequality (39) concludes that the trajectory tracking error r is uniformly bounded, and, more importantly, this bound can be explicitly calculated in the following. The Lyapunov function can be basically bounded as $\alpha_{1}\left(\left\|e\right\|\right)\leq V\leq \alpha_{2}\left(\left\|e\right\|\right)$ , where $\alpha_{1}\left(∙\right)$ and $\alpha_{2}\left(∙\right)$ are certain functions and will be defined later. Then, the ultimate bound $B_{e}$ on the tracking error e can be defined as

$$B_{e}=\alpha_{1}^{-1}\left(\alpha_{2}\left(\left\|z_{l}\right\|\right)\right)$$ (18)

where $z_{l}$ is the limiting term that satisfies $\dot{V}<0\forall \left\|e\right\|\geq z_{l}\geq 0$ .

Since the inertia matrix M is positive-definite and bounded, the subsequent inequality can be derived as

$$\frac{1}{2}\underline{M}{\left\|e\right\|}^{2}\leq V\leq \frac{1}{2}\bar{M}{\left\|e\right\|}^{2}$$ (19)

where $\underline{M}$ and $\bar{M}$ are the minimal and maximal eigenvalues of the inertia matrix M , respectively. It should be noticed that the left and right sides of (40) correspond to $\alpha_{1}\left(\left\|e\right\|\right)$ and $\alpha_{2}\left(\left\|e\right\|\right)$ , respectively. By adopting the right side of (39) as the limiting term $z_{l}$ and substituting the inequality (42) into (41), the bound on the tracking error can be calculated as

$$B_{e}=\sqrt{\frac{\bar{M}{\left\|\tilde{M}\dot{e}+\tilde{C}e-e_{d}\right\|}^{2}}{\underline{M}\theta^{2}{\underline{K}}_{D}^{2}}}$$ (20)

Noticeably, the feedback gain $K_{D}$ is included in the formulation of the bound $B_{e}$ , which means that the bound on the allowable trajectory tracking error can be manipulated by directly varying the value of $K_{D}$ , and the amount of robotic assistance can be consequently adjusted. Although adequately large values of $K_{D}$ results in minimal bound on tracking error, perfect tracking effect is not desirable to stimulate patients’ potential motor capabilities (Pehlivan et al., 2015). Appropriate allowable tracking error can facilitate improved rehabilitation efficacy, especially aiming to promote the patient’s active participation. The increased or decreased value of $K_{D}$ is suitable in the following practical situations.

***Situation 1:*** When the patient with severe motor disability has difficulty in completing the training task or learning a motion, increasing the value of $K_{D}$ leads to reduced allowable tracking error, and larger robotic actuation is provided for assistance.

***Situation 2:*** When the patient attempts to stimulate muscle strength to challenge themselves with more difficult tasks, decreasing the value of $K_{D}$ leads to increased allowable tracking error, and smaller robotic actuation is provided to spare more space for the patient’s effort.

Therefore, the selection of the feedback gain $K_{D}$ plays an important role in addressing the trade-off between accurate trajectory tracking and sufficient participation encouragement. To solve this problem, a feedback gain modification algorithm is put forward to render patients complete and even challenge the task according to their residual motor capabilities and motion intention.

A parameter $e^{*}$ is introduced to define the maximum allowable trajectory tracking error. The average tracking error in a certain task is recorded as $e_{i}$ with the feedback gain $K_{D,i}$ , which will be updated as $K_{D,i+1}$ in the next task based on the patient’s performance. The performance metric is the human-robot interaction torque to evaluate voluntary movement ability. The muscle activation in the current task can be normalized as $u_{i}=\left|\tau_{h,i}\right|/\left|{\hat{\tau }}_{h,i}\right|$ , where $\tau_{h,i}$ denotes the average interaction torque in the current task, and $\left|{\hat{\tau }}_{h,i}\right|$ denotes the human’s joint torque to complete the task in the absence of robotic assistance. Similarly, the human’s performance in the prior task can be expressed as $u_{i-1}=\left|\tau_{h,i-1}\right|/\left|{\hat{\tau }}_{h,i}\right|$ . Comparison of the human’s performance in the current task to the previous task is considered the variance tendency of the subject’s motor capability. Concretely, if $u_{i}<u_{i-1}$ , the patient shows a downward tendency in muscle strength stimulation, the future feedback gain $K_{D,i+1}$ should be increased to meet ***Situation 1***; otherwise, if $u_{i}>u_{i-1}$ , the patient shows an upward tendency in rehabilitation efficacy, and the future feedback gain $K_{D,i+1}$ should be decreased to meet ***Situation 2***. The updating of the feedback gain occurs at the end of each task trajectory, and it conforms to the following law

$$K_{D,i+1}=\left(1+\varpi_{i}\right)K_{D,i}$$ (21)

where $\varpi_{i}$ is the change rate and satisfies $-1<\varpi_{i}<1$ . Specifically, $\varpi_{i}\in \left(-1,0\right)$ means decreasing the future feedback gain with respect to the current one, whereas $\varpi_{i}\in \left(0,1\right)$ means increasing the tendency. The formulation of the change rate is defined as

$$\varpi_{i}=\frac{u_{i-1}-u_{i}}{\tau_{h,i}^{\exp}}∙{\left(\frac{u_{i}}{u_{i-1}}\right)}^{sign\left(u_{i-1}-u_{i}\right)}\varpi_{nom}$$ (22)

where $\varpi_{nom}$ is the nominal change rate and is predetermined as a constant to limit the maximal tracking error to less than $e^{*}$ . The sign of $\varpi_{i}$ depends on the variance tendency of the subject’s motor capability. For instance, if $u_{i}$ is larger than $u_{i-1}$ , $\varpi_{i}\in \left(-1,0\right)$ , the algorithm dictates that the subject has the potential to exhibit better performance in the next task, and the feedback gain decreases for larger error bound. Conversely, if $u_{i}$ is larger than $u_{i-1}$ , $\varpi_{i}\in \left(0,1\right)$ , the algorithm dictates that the subject fails to complete the current task with improved voluntary muscle strength, and the feedback gain increases for more assistance in the next task. The magnitude of $\varpi_{i}$ is decided by both the maximum tracking error and the performance variance. 

Combining the AAN controller (17) with the feedback gain modification algorithm (21) and (22), the control framework can provide a highly efficient and autonomous training strategy for robot-assisted rehabilitation.

## 5 Experiments

In order to validate the proposed control framework, the lower extremity rehabilitation robot mentioned in Xu et al. (2019), Xu et al. (2021) was utilized to conduct a series of experiments. The experiments were carried out on three healthy subjects. All subjects were informed of the detailed operation procedures and potential risks and signed consent forms before participation. The experiments were approved by the ethics committee of Hefei Institutes of Physical Science, Chinese Academy of Sciences (approval number: IRB-2019-0018). Two DOFs of the robot, including hip flexion/extension and knee flexion/extension, were involved in the training. Before operation, the reference trajectories were prescribed by physiotherapists to ensure rhythmic and comfortable training motion, and they were then fed into the robot controller. During the training, the subjects were asked to track the reference trajectories with the assistance of the rehabilitation robot actuation. Once the interaction force exerted by the subjects was detected by sensors, the reference trajectory was deformed to generate another optimal desired trajectory, and the robot was controlled to cooperate with the subject to complete the modified task motion. It should be noted that the subjects were not allowed to voluntarily move in the opposite direction from the task trajectory for accurate calculation of the deformed trajectory and safety guarantee.

**FIGURE 4:**
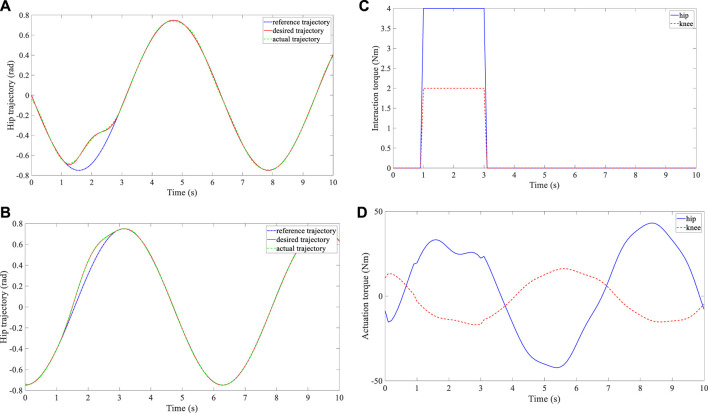
Experimental results of subject 1. **(A)** Trajectory deformation and tracking of the hip joint. **(B)** Trajectory deformation and tracking of the knee joint. **(C)** Interaction torque at the hip and knee joints. **(D)** Actuation torque at the hip and knee joints.

**FIGURE 5:**
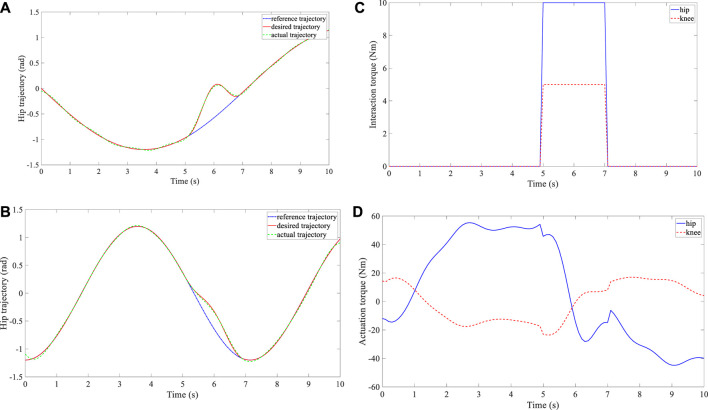
Experimental results of subject 2. **(A)** Trajectory deformation and tracking of the hip joint. **(B)** Trajectory deformation and tracking of the knee joint. **(C)** Interaction torque at the hip and knee joints. **(D)** Actuation torque at the hip and knee joints.

**FIGURE 6:**
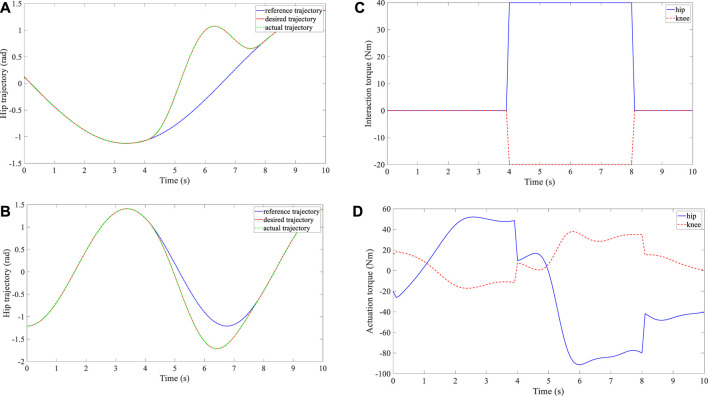
Experimental results of subject 3. **(A)** Trajectory deformation and tracking of the hip joint. **(B)** Trajectory deformation and tracking of the knee joint. **(C)** Interaction torque at the hip and knee joints. **(D)** Actuation torque at the hip and knee joints.

Three groups of experiments were performed for the three subjects, and three different reference trajectories were configured. The time interval between the consecutive waypoints was set at δ=0.01 s , and the prediction vector of the interaction torque was set at $\beta =\vec{1}$ . The vector field function was updated four times for hip flexion, hip extension, knee flexion, and knee extension in one walking cycle, where the computation efficiency was adequate to ensure instantaneous and accurate trajectory deformation. The amount and variance of pHRI differed across the three subjects due to their individual motor capabilities and motion intentions. The experimental results are shown in Figures 4–6. The subfigures A and B demonstrate the trajectory deformation and tracking of the hip and knee joint, respectively. The variance of the interaction torque at the hip and knee joints is illustrated in subfigure C, and the robotic actuation torque is presented in subfigure D. The experimental results indicate that the proposed trajectory generator can continuously produce a smooth and optimal desired trajectory once the subject exerts force on the robot. When the interaction torque disappears, the desired trajectory gradually converges back to the predetermined reference trajectory. In this regard, the shared control between the robot’s desired trajectory and the human’s voluntary effort is realized. Additionally, based on the proposed AAN controller, the actual trajectory output from the robot actuation can track the desired trajectory well.

In order to exhibit the control performance more intuitively, quantitative evaluation with three metrics was conducted. In terms of trajectory smoothness, the dimensionless squared jerk (Hogan and Sternad, 2009) was adopted, and its definition is presented in (23). A smaller DSJ value indicates a smoother movement trajectory. As for the compliance assessment, the energy per unit distance (EPUD) (Lee et al., 2018) was selected as (24). When improved compliance was shown, the subject could drive the robot with less interaction torque. A smaller EPUD value indicates higher robot compliance. The root mean square error (RMSE) defined in (25) was utilized to reveal the position error between the desired trajectory and actual trajectory. A smaller value of RMSE indicates better tracking effect. The three metrics are formulated as follows.

$$\text{DSJ}=\int_{t_{a}}^{t_{b}}{\overset{\ldots }{q}\left(t\right)}^{2}dt\frac{{\left(t_{b}-t_{a}\right)}^{5}}{q_{\max}^{2}}$$ (23)

$$\text{EPUD}=\frac{\sum_{j=1}^{N}\left|\tau_{h}\left(t_{j}\right)∆d\left(t_{j}\right)\right|}{\sum_{j=1}^{N}\left|∆d\left(t_{j}\right)\right|}$$ (24)

$$\text{RMSE}=\sqrt{\frac{1}{N}\sum_{j=1}^{N}{\left(q\left(t_{j}\right)-q_{d}\left(t_{j}\right)\right)}^{2}}$$ (25)

In (23), the parameters $t_{a}$ and $t_{b}$ are the start and end time of the trajectory, $q_{\max}$ is the maximum amplitude of the trajectory, and $\overset{\ldots }{q}\left(t\right)$ is the third time-derivative of the trajectory. In (24), j=1,2,…,N is the sample number, $\tau_{h}\left(t_{j}\right)$ is the human-robot interaction torque at the time $t_{j}$ , and $∆d\left(t_{j}\right)$ is the deviation between the reference trajectory and desired trajectory at the time $t_{j}$ . In (25), $q\left(t_{j}\right)$ and $q_{d}\left(t_{j}\right)$ are actual and desired trajectory at the time $t_{j}$ , respectively.

Additionally, to better manifest the advantage of the proposed control framework, comparison experiments were performed with an admittance control without trajectory deformation and feedback gain modification algorithms (Li et al., 2017). The admittance control was implemented with the same robot and subject. The admittance parameters were regulated while responding to the subjects’ biological actions. The trajectory deformation and tracking performance of both control systems were evaluated with the abovementioned three metrics. Additionally, in order to evaluate rehabilitation efficacy, muscle activation improvement was normalized and recorded, and the Fugl-Meyer assessment (FMA) was also deployed for clinical evaluation. Higher normalized EMG value and FMA score indicate rehabilitation improvement. Each trial was conducted three times for accuracy, and the mean values of these metrics are recorded in Table 1. The robot motion compliance and movement smoothness of the hip and knee joint trajectories generated by the deformation trajectory algorithm are much better than those with the admittance control. Furthermore, the tracking performance under the feedback gain algorithm proved more satisfactory compared to the admittance control. The enhancement of the muscle strength and clinical assessment scores is evident compared with the performance without the proposed control framework. Overall, the comparison results prove that the control framework can effectively help the patient learn to move in the proper trajectory, and the training becomes more challenging and brings better rehabilitation efficacy.

**TABLE 1:** Comparison results.

Control strategy	Trajectory				Trajectory tracking		Muscle activation		Clinical assessment
	Deformation								
	DSJ		EPUD		RMSE (rad)		Normalized EMG values		FMA
	Hip	Knee	Hip	Knee	Hip	Knee	Hip	Knee	
Proposed Control	7.66 × 10^8^	3.1 ×	13.24	13.58	0.07	0.05	0.87	0.91	30.4
		10^8^							
Admittance Control	3.78 × 10^12^	1.24 ×	16.37	16.61	0.19	0.16	0.48	0.53	18.8
		10^12^							

**FIGURE 7:**
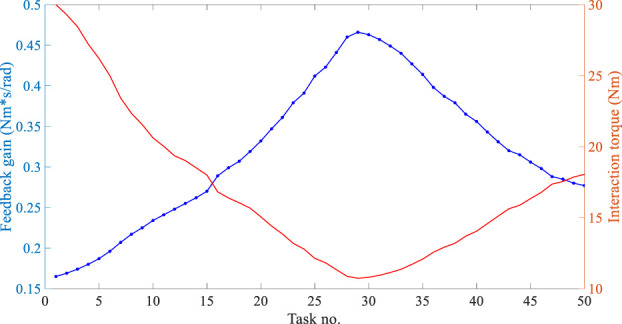
Experimental results of the Feedback gain modification algorithm.

Next, the feedback gain modification algorithm for the AAN controller was experimentally examined during the optimized training task. The subjects were required to voluntarily exert forces on the robot, and the feedback gain $K_{D}$ was adapted according to the subjects’ performance, producing subject-adaptive robotic assistance. At the end of each training task, questionaries were set and filled in to identify whether the current task was easier or more difficult compared to the previous task. The questionnaire responses only helped assessments of the pilots’ subjective intention without affecting the robot controller. After that, the subsequent training task was operated immediately. The tracking performance of the AAN controller, variance of the feedback gain $K_{D}$ , and the human-robot interaction torque were measured in real time and are depicted in Figure 7. It can be seen from the figure that the feedback gain and tracking error are functions of the interaction torque. The experimental results demonstrate that the feedback gain $K_{D}$ can respond correctly to the subjects’ motor capabilities. When subjects complete the task with more active involvement, $K_{D}$ decreases and the allowable trajectory tracking error increases, and the magnitude of robotic assistance decreases for further encouragement, and *vice versa*. The variance tendency of $K_{D}$ is consistent with the questionnaire results. Furthermore, no matter how the tracking error varies, the user-selected maximum allowable trajectory tracking error $r^{*}$ is always larger than or equal to the error in each task. Therefore, it can be concluded that the feedback gain modification algorithm can effectively adjust the robotic assistance according to the subjects’ changing performance, hopefully encouraging impaired patients’ active participation and facilitating rehabilitation efficacy.

## 6 Conclusion

In this paper, a control framework is proposed for the simultaneous adaptation of training tasks and robotic assistance for robot-assisted rehabilitation. Specifically, a trajectory deformation algorithm is developed to enable pHRI to regulate the task difficulty in real time, generating a smooth and compliant desired trajectory. Furthermore, an AAN controller, along with a feedback gain modification algorithm, is designed to motivate patients’ active participation, where the robotic assistance is adjusted by evaluating the patients’ performance variance and determining the trajectory tracking error bound. Appropriate training difficulty and assistance level are two important issues in robot-assisted rehabilitation. In this study, the appropriate training difficulty is expressed in the form of making a proper trajectory, which is realized with the proposed trajectory deformation algorithm; and the appropriate assistance level is expressed in the form of increasing the user’s EMG level, which is realized with the proposed AAN controller with the feedback gain modification algorithm. The balance between these two issues is essential for better rehabilitation efficacy, and the proposed control framework can address this balance well. A lower extremity rehabilitation robot with MR actuators is then employed to validate the effectiveness of the proposed control framework. Experimental results demonstrate that the training task difficulty and robotic assistance level can be regulated appropriately according to subjects’ changing motor capabilities.

In future work, more novel methods will be explored to estimate human motor capabilities and improve pHRI control strategies. More diverse training tasks will be involved to meet the rehabilitation requirements of different degrees and types of impairments. Machine learning may be adopted to ensure better time efficiency and greater adaptability of robotic assistance modification. Furthermore, more clinical trials will be carried out to expand the proposed control framework into clinical application.

## Data Availability

The raw data supporting the conclusions of this article will be made available by the authors, without undue reservation.

## Appendix

### AppendixDetermination of Vector Field Function

As presented in (9), the vector field function $\Phi \left(t\right)$ is applied to shape the deformed trajectory, and its determination should follow the subsequent rules.

***Rule 1:* Continuity.** Once the human force $\tau_{h}$ is exerted at $t_{i}$ , the predefined reference trajectory $q_{d}^{*}$ starts to be deformed to $q_{d}$ ; after time $t_{f}$ , the robot again follows its reference trajectory $q_{d}^{*}$ . Hence, in order to ensure the continuous transitioning between the original and deformed trajectories, the field vector function $\Phi \left(t\right)$ and its time-differential $\dot{\Phi }\left(t\right)$ is constrained as

$$\Phi \left(t_{i}\right)=\Phi \left(t_{f}\right)=0$$

$$\dot{\Phi }\left(t_{i}\right)=\dot{\Phi }\left(t_{f}\right)=0$$
(26)

Then, the trajectory configuration on the boundary condition can be satisfied, i.e., $\gamma_{d}\left(t\right)={\tilde{\gamma }}_{d}\left(t\right)$ and ${\dot{\gamma }}_{d}\left(t\right)={\dot{\tilde{\gamma }}}_{d}\left(t\right)$ at both the start $t_{i}$ and the end $t_{f}$ .

Specifically, the deformed trajectory between $t_{i}$ and $t_{f}$ can be evenly divided into an arbitrary number of waypoints. We define the number of waypoints as N and the time interval between consecutive waypoints as δ . As introduced before, the time duration of pHRI is p , such that the number of waypoints along $\gamma_{d}$ and ${\tilde{\gamma }}_{d}$ can be calculated as $N=\frac{p}{\delta }+1$ .

Consequently, the original and deformed desired trajectory within the time interval $\left[t_{i},t_{f}\right]$ can be written as

$$\gamma_{d}=\left[q_{d}^{*}\left(t_{i}\right),q_{d}^{*}\left(t_{i}+\delta \right)\ldots \;q_{d}^{*}\left(t_{f}-\delta \right),q_{d}^{*}\left(t_{f}\right)\right]$$

$${\tilde{\gamma }}_{d}=\left[\Gamma_{d}^{s}\left(t_{i}\right),\Gamma_{d}^{s}\left(t_{i}+\delta \right)\ldots \Gamma_{d}^{s}\left(t_{f}-\delta \right),\Gamma_{d}^{s}\left(t_{f}\right)\right]$$

Applying this waypoint parameterization, the continuity statement (26) can be rewritten as

$$\left\{\begin{array}{c} \Gamma_{d}^{s}\left(t_{i}\right)-q_{d}^{*}\left(t_{i}\right)=\Phi \left(t_{i}\right)=0\\ \Gamma_{d}^{s}\left(t_{i}+\delta \right)-q_{d}^{*}\left(t_{i}+\delta \right)=\Phi \left(t_{i}+\delta \right)=0\\ \Gamma_{d}^{s}\left(t_{f}-\delta \right)-q_{d}^{*}\left(t_{f}-\delta \right)=\Phi \left(t_{f}-\delta \right)=0\\ \Gamma_{d}^{s}\left(t_{f}\right)-q_{d}^{*}\left(t_{f}\right)=\Phi \left(t_{f}\right)=0 \end{array}\right.$$

The above equation can be further rewritten as

$$B\left({\tilde{\gamma }}_{d}-\gamma_{d}\right)=B\Phi =0$$
(27)

where

$$B=\left[\begin{array}{ccccccc} 1 & 0 & 0 & \cdots & 0 & 0 & 0\\ 0 & 1 & 0 & \cdots & 0 & 0 & 0\\ 0 & 0 & 0 & \cdots & 0 & 1 & 0\\ 0 & 0 & 0 & \cdots & 0 & 0 & 1 \end{array}\right]\in R^{4\times N}$$

***Rule 2:* Smoothness.** Although the reference trajectory $q_{d}^{*}$ has been set to be as smooth as possible through modeling from the healthy subject gait database, the naturality of the deformed desired trajectory $q_{d}$ should also be maintained to guarantee the patient’s comfort and security. Numerous observations have demonstrated that the healthy human’s movement complies well with the minimum-jerk model (Li et al., 2017). Similarly, the minimum-jerk deformed trajectory can be derived by satisfying the vector field function as

$$\overset{\ldots }{\Phi }=\delta^{-3}∙A\Phi$$
(28)

where

$$A=\left[\begin{array}{ccccc} 1 & 0 & 0 & \ldots & 0\\ -3 & 1 & 0 & \ldots & 0\\ 3 & -3 & 1 & \ldots & 0\\ -1 & 3 & -3 & \ldots & 0\\ 0 & -1 & 3 & \ldots & 0\\ 0 & 0 & -1 & \ldots & 0\\ \vdots & \vdots & \vdots & \ddots & \vdots \\ 0 & 0 & 0 & \ldots & 1\\ 0 & 0 & 0 & \ldots & -3\\ 0 & 0 & 0 & \ldots & 3\\ 0 & 0 & 0 & \ldots & -1 \end{array}\right]\in R^{\left(N+3\right)\times N}$$

***Rule 3:* Compliance.** It is pHRI that initiates the trajectory deformation and decides the deformed trajectory shape; the vector field function $\Phi \left(t\right)$ is highly correlated with the interaction torque $\tau_{h}$ . Analogous to (Losey and O’Malley, 2018), a cost function is formulated to reveal the variation of the trajectory deformation energy as

$$J\left({\tilde{\gamma }}_{d}\right)={\left({\tilde{\gamma }}_{d}-\gamma_{d}\right)}^{T}\left(-\beta \tau_{h}\left(t_{i}\right)\right)+\frac{1}{2\alpha }{\left({\tilde{\gamma }}_{d}-\gamma_{d}\right)}^{T}\left(A^{T}A\right)\left({\tilde{\gamma }}_{d}-\gamma_{d}\right)$$
(29)

where α is a positive constant and ${\hat{\tau }}_{h}$ is the prediction of the human-robot interaction torque. It should be noticed that the trajectory deformation occurs at the current time $t_{i}$ when the human first interacts with the robot and results in the interaction torque $\tau_{h}\left(t_{i}\right)$ . Since the future interaction torque values are required to compute the energy of the trajectory deformation but remain unknown, an online prediction of the future interaction torque is developed as $\beta \tau_{h}\left(t_{i}\right)$ , where $\beta \in R^{N}$ is the prediction vector.

The proposed cost function (29) contains two terms: the first term means the work done by the trajectory deformation to the human; and the second term is the squared norm of Φ with respect to the finite differencing matrix $A^{T}A$ (*A* is mentioned in (28)) to ensure the smoothness and naturality of the deformed trajectory.

In order to ensure the compliance of the deformed trajectory, the cost function (29) should be minimized under the constraint (27) to optimize the value of the field vector function Φ . The optimization problem can be formulated as

$$\text{minimize }J\left({\tilde{\gamma }}_{d}\right)$$

$$\text{subject to }B\left({\tilde{\gamma }}_{d}-\gamma_{d}\right)=0$$
(30)

A Lagrangian function is defined as follows to solve the above optimization problem.

$$L\left({\tilde{\gamma }}_{d},\lambda \right)=J\left({\tilde{\gamma }}_{d}\right)+\lambda^{T}B\left({\tilde{\gamma }}_{d}-\gamma_{d}\right)$$
(31)

where $\lambda \in R^{4}$ is a vector of Lagrange multipliers. By calculating the partial derivative of (31), we have

$$\partial_{{\tilde{\gamma }}_{d}}L\left({\tilde{\gamma }}_{d},\lambda \right)=-\beta \tau_{h}\left(t_{i}\right)+\frac{1}{\alpha s}A^{T}A\left({\tilde{\gamma }}_{d}-\gamma_{d}\right)+B^{T}\lambda =0$$

$$\partial_{\lambda }L\left({\tilde{\gamma }}_{d},\lambda \right)=B\left({\tilde{\gamma }}_{d}-\gamma_{d}\right)=0$$
(32)

Through further computation after (32), the subsequent equation (33) can be obtained to reveal the relationship between the field vector function and the interaction torque. In addition to Lagrange multipliers, the reader can refer to (Wu et al., 2020) to find another solver, i.e., linear variational inequality-based primal-dual neural network.

$$V=\rho G\beta \tau_{h}\left(t_{i}\right)$$
(33)

where

$$G=\left(I-{\left(A^{T}A\right)}^{-1}B^{T}{\left(B{\left(A^{T}A\right)}^{-1}B^{T}\right)}^{-1}B\right){\left(A^{T}A\right)}^{-1}$$
(34)

where $I\in R^{N\times N}$ is an identity matrix.

The determination of the parameter ρ in (33) has significant impacts on the shape of Φ . In robotics-assisted rehabilitation, the duration time of the human-robot interaction is relatively long because the patient mostly tries to participate actively to guide the robot, which is different from the statement in (Damsgaard et al., 2006). Considering the above factors, the parameter ρ is defined as

$$\rho =\mu \delta \frac{G}{\left(p+\delta \right)\left\|G\right\|}$$
(35)

where μ denotes the level of assistance, which regulates whether it is the robot or human that the trajectory deformation arbitrates toward.

Therefore, following the rule of continuity, smoothness, and compliance, the ultimate expression of the vector field function is clarified as

$$\Phi \left(t\right)=\mu \delta H\beta \tau_{h}\left(t_{i}\right)$$
(36)

where

$$H=\frac{G}{\left(p+\delta \right)\left\|G\right\|}$$

### 7.2 Stability Analysis of AAN Controller

Combining the modified robot dynamics (15), error dynamics (16), and AAN controller (17), the following equation can be yielded.

$$\hat{M}\dot{e}+\hat{C}e+K_{D}e+\tau_{d}=0$$
(37)

For stability analysis, consider a Lyapunov candidate function as

$$V=\frac{1}{2}e^{T}Me$$
(38)

Then, the time-derivative of the Lyapunov function is

$$\dot{V}=-e^{T}K_{D}e+e^{T}\left(\tilde{M}\dot{e}+\tilde{C}e-\tau_{d}\right)$$
(39)

Let us introduce a constant $\theta \in \left(0,1\right)$ ; the time-derivative of the Lyapunov function can be further written as

$$\dot{V}\leq -{\underline{K}}_{D}{\left\|e\right\|}^{2}+\left\|e\right\|∙\left\|\tilde{M}\dot{e}+\tilde{C}e-\tau_{d}\right\|\leq \left(\theta -1\right){\underline{K}}_{D}{\left\|e\right\|}^{2}-\theta {\underline{K}}_{D}{\left\|e\right\|}^{2}+\left\|e\right\|∙\left\|\tilde{M}\dot{e}+\tilde{C}e-\tau_{d}\right\|$$
(40)

Hence, if the following inequality is satisfied,

$$\left\|e\right\|\geq \frac{\left\|\tilde{M}\dot{e}+\tilde{C}e-\tau_{d}\right\|}{\theta {\underline{K}}_{D}}$$
(41)

The time-derivative of the Lyapunov function satisfies

$$\dot{V}\leq \left(\theta -1\right){\underline{K}}_{D}{\left\|e\right\|}^{2}<0$$
(42)

Through the stability analysis, the Lyapunov candidate V≥0 and its time-derivative $\dot{V}<0$ are ensured, and it can be concluded that the proposed control system yields a tracking error with uniformly ultimately bounded stability.
